# A stabilized finite element method for finite-strain three-field poroelasticity

**DOI:** 10.1007/s00466-017-1381-8

**Published:** 2017-03-01

**Authors:** Lorenz Berger, Rafel Bordas, David Kay, Simon Tavener

**Affiliations:** 1 Innersight Labs, 7 Astbury House, Lambeth Walk, London, SE11 6LZ UK; 2 Roxar Ltd, Emerson Process Management, Northbrook House, Oxford Science Park, Oxford, OX4 4GA UK; 3 Department of Computer Science, University of Oxford, Wolfson Building, Parks Road, Oxford, OX1 3QD UK; 4 Department of Mathematics, 100 Statistics Building, Colorado State University, Fort Collins, CO 80523 USA

## Abstract

We construct a stabilized finite-element method to compute flow and finite-strain deformations in an incompressible poroelastic medium. We employ a three-field mixed formulation to calculate displacement, fluid flux and pressure directly and introduce a Lagrange multiplier to enforce flux boundary conditions. We use a low order approximation, namely, continuous piecewise-linear approximation for the displacements and fluid flux, and piecewise-constant approximation for the pressure. This results in a simple matrix structure with low bandwidth. The method is stable in both the limiting cases of small and large permeability. Moreover, the discontinuous pressure space enables efficient approximation of steep gradients such as those occurring due to rapidly changing material coefficients or boundary conditions, both of which are commonly seen in physical and biological applications.

## Introduction

Poroelasticity theory assumes a superposition of solid and fluid components to capture complex interactions between a deformable porous medium and the fluid flow within it, and was originally developed to study geophysical applications such as reservoir geomechanics [26, 28, 41]. Fully saturated, incompressible poroelastic models have since been used to model a variety of biological tissues and processes. Biological examples include the coupling of flow in coronary vessels with the mechanical deformation of myocardial tissue to create a poroelastic model of coronary perfusion [13, 15]. Other examples include modelling tissue deformation and the ventilation in the lungs [6], protein-based hydrogels embedded within cells [22], brain oedema and hydrocephalus [39, 56], microcirculation of blood and interstitial fluid in the liver lobule [36], and interstitial fluid and tissue in articular cartilage and intervertebral discs [21, 24, 42].

When using the finite element method to solve the poroelastic equations the main challenge is to ensure convergence of the method and prevent numerical instabilities that often manifest themselves in the form of spurious oscillations in the pressure field. It has been suggested that this problem is caused by the saddle point structure in the coupled equations resulting in a violation of the famous Ladyzhenskaya–Babuska–Brezzi (LBB) condition, thus highlighting the need for a stable combination of mixed finite elements [23].

In addition, there is a need for methods that do not give rise to localised pressure oscillations when seeking to approximate steep pressure gradients in the solution. For example, when modelling the diseased lung, abrupt changes in tissue properties and heterogeneous airway narrowing are possible. This can result in a patchy ventilation and pressure distribution [51]. In this situation methods that solve the poroelastic equations using a continuous pressure approximation struggle to capture the steep gradients in pressure and produce localised oscillations in the pressure [46]. Steep pressure gradients can also result from imposed Dirichlet pressure boundary conditions such as those in Terzaghi’s problem [43, 54]. The method presented here is able to overcome these types of pressure instability.

### Two variable versus three variable formulations

The linear (infinitesimal strain) poroelastic equations are often solved in a displacement and pressure formulation from which the fluid flux can be recovered [43, 54]. The stability and convergence of this displacement and pressure $(\varvec{u}/p)$ formulation was analysed in [43] and error bounds for inf-sup stable combinations of finite element spaces (e.g. Taylor-Hood elements) were obtained. In the current work we maintain the fluid flux as a variable, resulting in a three-field, displacement, fluid flux and pressure formulation. Retaining the fluid flux as a primary variable has the following advantages.

1. It allows for greater accuracy in the fluid velocity field. This can be of particular interest when a poroelastic model is coupled with an advection diffusion equation, e.g., to account for gas exchange, thermal effects, contaminant transport or the transport of nutrients or drugs within a porous tissue [30].
2. Physically meaningful boundary conditions can be applied at the interface when modelling the interaction between a fluid and a poroelastic structure [5].
3. It allows for an easy extension of the fluid model from a Darcy to a Brinkman flow model, for which there are numerous applications in modelling biological tissues [30].
4. It avoids the calculation of the fluid flux in post-processing.

A clear disadvantage of a three-field formulation is the increased number of degrees of freedom of the linear system arising from the FEM discretisation, although this difficulty is mitigated with the proposed element, see comment 7 in §1.4.

### Previous results: infinitesimal strain

Error estimates for finite element solutions of the linear three-field problem, using continuous piecewise linear approximations for displacements and mixed low-order Raviart–Thomas elements for the fluid flux and pressure variables, are presented in [44, 45]. However this method was found to be susceptible to spurious pressure oscillations [47]. In an effort to overcome these pressure oscillations, a discontinuous linear three-field method was analysed in [38] with moderate success, and a linear non-conforming three-field method was analysed in [58]. However no implementation of these methods in 3D has yet been presented.

Due to the size of the discrete systems resulting from a three field approach, there has been considerable work on operating splitting (iterative) approaches in which the poroelastic equations are separated into a fluid problem and deformation problem [19, 28, 32, 53]. These methods are often able to take advantage of existing finite element software for elasticity and fluid flow. Matrix assembly for discontinuous and non-conforming finite elements in 3D can be complicated and calculating stresses using these methods can be particularly challenging. Methods that use standard and simple to implement elements are very appealing [55]. In [28], a linear three-field mixed finite element method using lowest order Raviart–Thomas elements was shown to overcome Dirichlet boundary type pressure instabilities.

The finite volume method has been used by [31, 32] to discretize the flow. This results in a discontinuous approximation of the fluid pressure which is able to overcome localised pressure oscillations due to steep pressure gradients in the solution.

Introducing a displacement stress field, e.g. see [49], reduces the regularity requirements on the displacement field, thereby allowing for the implementation of a four-field conforming Raviart–Thomas element, but consequently greatly increasing the overall size and complexity of the problem.

### Previous results: finite strain

Monolithic approaches for solving the quasi-static two-field incompressible finite-strain deformation equations are outlined in [1]. Two different approaches are advocated, a mixed-penalty formulation, in which the continuity condition is imposed using a penalty approach, and a mixed solid velocity–pressure formulation, where the linear momentum for the fluid is used to eliminate the fluid velocity in the remaining equations. The solid velocity–pressure formulation is similar to the commonly used reduced $(\varvec{u}/p)$ formulation in [4]. Two-field formulations require a stable mixed element pair such as the popular Taylor-Hood element to satisfy the LBB inf-sup stability requirement. However, using a continuous pressure element means that jumps in material coefficients may introduce large solution gradients across the interface, requiring severe mesh refinement or failing to reliably capture jumps in the pressure solution [54]. An operator splitting (iterative) approach for a near incompressible model is described by [13].

A three-field (displacement, fluid flux, pressure) formulation has been outlined in [37], however this method uses a low-order mixed finite element approximation without any stabilisation and therefore is not inf-sup stable. A three-field finite element using a continuous pressure approximation has been implemented in [52].

For both two-field and three-fields formulations and for any choices of finite element, implementation, construction and linearization of the nonlinear equations and convergence of the nonlinear mechanics problem using Newton’s method or other iterative procedure is also nontrivial [50].

### Contributions of the current work

In [7], we developed a stabilized, low-order, three-field mixed finite element method for the fully saturated, incompressible, small deformation case for which a linearly elastic model is sufficient. Low-order finite element methods are relatively easy to implement and allow for efficient preconditioning [20, 27, 55]. Rigorous theoretical results for the stability and optimal convergence rate for linear poroelasticity were presented in [7]. The stabilization term requires only a small amount of additional computational work and can be assembled locally on each element using standard finite element information, leading to a symmetric addition to the original system matrix and preserving any existing symmetry. The effect of the stabilization on the conservation of mass is minimal in 3D, and decreases as the mesh is refined, see [7].

In the current work we present a monolithic mixed finite element method to solve the fully incompressible three-field finite-strain poroelasticity equations. We use a low order approximation, namely, continuous piecewise-linear approximation for the displacements and fluid flux, and piecewise-constant approximation for the pressure. The finite-strain case is a non-trivial extension of the infinitestimal-strain implementation and requires several challenges to be overcome including: (1) problem formulation and construction of the weak equations, (2) accurate integration over the deformed domain, (3) linearization of the weak equations, (4) construction and convergence of a Quasi-Newton iteration, and (5) time step selection. The main contributions of this work are therefore as follows.

1. A method to solve finite-strain fully incompressible poroelasticity using a stabilized discontinuous pressure approximation. [Note that for the linear (infinitesimal strain) equations other methods that use a discontinuous pressure approximation have been previously presented [7, 28, 38, 58].]
2. A method for finite-strain fully incompressible poroelasticity that is both inf-sup stable and is able to overcome localized pressure oscillations.
3. A finite element method that is robust within all modelling regimes. Large differences in permeability within the computational domain can result in regions in which Darcy flow dominates over elastic effects and regions in which elastic effects are dominant. This low-order element is reliable in both scenarios, providing an effective numerical approach for problems in which heterogeneity presents computational challenges.
4. A finite element method that results in a discrete system with blocks arising from simple linear finite elements allowing fast solver approaches and preconditioning techniques to be easily implemented.
5. A finite element method for a finite-strain poroelastic model that resolves steep pressure gradients without localized oscillations.

We present a quasi-static finite-strain incompressible poroelastic model in Sect. 2 and develop the stabilized nonlinear finite-element method in Sect. 3. Implementation details are provided in Sect. 4 and in the appendices. In Sect. 5, we present a range of numerical experiments to verify the accuracy of the method and to demonstrate its ability to reliably capture steep pressure gradients.

## Poroelasticity theory

Two complementary approaches have been developed for modelling a deformable porous medium. Mixture theory, also known as the Theory of Porous Media (TPM) [8, 10, 11], has its roots in the classical theories of gas mixtures and makes use of a volume fraction concept in which the porous medium is represented by spatially superposed interacting media. An alternative, purely macroscopic approach is mainly associated with the work of Biot. A comprehensive development of the macroscopic theory appears in [16]. Relationships between the two theories are explored in [14, 17]. As is most common in biological applications, we use the mixture theory for poroelasticity as outlined in [8] and recently summarized in [52].

### Kinematics

**Fig. 1:**
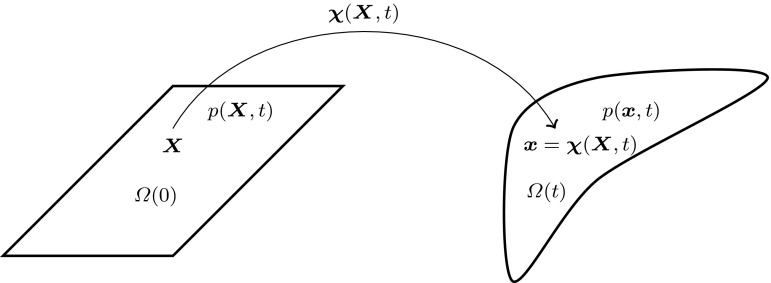
Illustration of the solid deformation

Let the volume $\varOmega (0)$ be the undeformed Lagrangian (material) reference configuration and let $\varvec{X}$ indicate the position of a particle in $\varOmega (0)$ at $t=0$. The position of a particle in the deformed configuration $\varOmega (t)$ at time $t > 0$ is given by $\varvec{x}$, with $\varvec{x}=\varvec{\chi }(\varvec{X},t)$ as shown in Fig. 1. The deformation map, $\varvec{\chi }(\varvec{X},t)$, is a continuously differentiable, invertible mapping from $\varOmega (0)$ to $\varOmega (t)$. Thus the inverse of the deformation map, $\varvec{\chi }^{-1}(\varvec{x},t)$, is such that $\varvec{X}=\varvec{\chi }^{-1}(\varvec{x},t)$. The displacement field is given by

1 $$\begin{aligned} \varvec{u}(\varvec{X},t)=\varvec{\chi }(\varvec{X},t)-\varvec{X}. \end{aligned}$$

The deformation gradient tensor is

2 $$\begin{aligned} \varvec{F}=\frac{\partial \varvec{\chi }(\varvec{X},t)}{\partial \varvec{X}}, \end{aligned}$$

and the symmetric right Cauchy–Green deformation tensor is

3 $$\begin{aligned} \varvec{C}={\varvec{F}}^{T}{\varvec{F}}. \end{aligned}$$

The Jacobian is defined as

4 $$\begin{aligned} J=\text{ det }(\varvec{F}), \end{aligned}$$

and represents the change in an infinitesimal control volume from the reference to the current configuration, i.e.,

5 $$\begin{aligned} d\varOmega (t)=Jd\varOmega (0). \end{aligned}$$

Note that $$J > 0$$.

### Volume fractions

We will only consider saturated porous media in which the fluid accounts for volume fractions $\phi _{0}(\varvec{X},t=0)$ and $\phi (\varvec{x},t)$ of the total volume in the reference and current configurations respectively, where $\phi$ is known as the porosity, defined through the Jacobian of the deformation (7). The fractions for the solid (or skeleton) are therefore $1-\phi _{0}$ and $1-\phi$ in the reference and current configuration, respectively. For the mixture, $\rho$ is the density in the current configuration given by

6 $$\begin{aligned} \rho =\rho ^{s}(1-\phi ) + \rho ^{f}\phi \quad \text{ in } \; \varOmega (t), \end{aligned}$$

where $\rho ^{s}$ and $\rho ^{f}$ are the densities of the fluid and solid, respectively. We assume that both the solid and the fluid are incompressible so that $\rho ^{s}=\rho ^{s}_{0}$ and $\rho ^{f}=\rho ^{f}_{0}$. Although both the solid and fluid are assumed to be incompressible, the control volume can expand or contract due to fluid entering or leaving the region, and

7 $$\begin{aligned} J = \frac{1-\phi _{0}}{1-\phi }. \end{aligned}$$

### The model

We define the boundary $\partial \varOmega (t) = \varGamma _{d}(t) \cup \varGamma _{n}(t)$ for the mixture and $\partial \varOmega (t) = \varGamma _{p}(t) \cup \varGamma _{f}(t)$ for the fluid, with an outward pointing unit normal $\varvec{n}$. We seek deformation $\varvec{\chi }(\varvec{X},t)$, fluid flux $\varvec{z}(\varvec{x},t)$ and pressure $p(\varvec{x},t)$ such that

8 $$\begin{aligned} \left. \begin{array}{cc} \begin{aligned} -\nabla \cdot ( \varvec{\sigma }_{e} -p\varvec{I}) &{}= \rho \varvec{f} \\ {\varvec{k}^{-1}\varvec{z}} + \nabla p &{}= \rho ^{f}\varvec{f} \\ \nabla \cdot (\varvec{\chi }_t + \varvec{z} ) &{}= g \\ {\varvec{\chi }}(\varvec{X},t)|_{\varvec{X}={\varvec{\chi }}^{-1}(\varvec{x},t)} &{}= \varvec{X} + \varvec{u}_{D} \\ (\varvec{\sigma }_{e}-p\varvec{I})\varvec{n} &{}= \varvec{t}_{N} \\ \varvec{z} \cdot \varvec{n} &{}= {q_{D}} \\ p &{}= p_{D} \\ {\varvec{\chi }}(\varvec{X},0) &{}= \varvec{X} \end{aligned}&{} \begin{aligned} &{}\text{ in } \; \varOmega (t), \\ &{}\text{ in } \; \varOmega (t), \\ &{}\text{ in } \; \varOmega (t), \\ &{}\text{ on } \; \varGamma _{d}(t), \\ &{}\text{ on } \; \varGamma _{n}(t), \\ &{}\text{ on } \; \varGamma _{f}(t), \\ &{}\text{ on } \; \varGamma _{p}(t), \\ &{}\text{ in } \; \varOmega (0). \end{aligned}\end{array} \right\} \end{aligned}$$

The fluid flux $\varvec{z}=\phi (\varvec{v}^{f}-\varvec{v}^{s})$ where $\varvec{v}^{f}$ and $\varvec{v}^{s}$ are the velocities of the fluid and solid components respectively, $\varvec{\chi }_t$ denotes $\frac{\partial \varvec{\chi }(\varvec{X},t)}{\partial t}$, $\varvec{u}_{D}, \varvec{q}_{D}, \varvec{p}_{D}$ are given boundary conditions, $\varvec{f}$ is a general external body force, *g* is a general source or sink term and $\varvec{\sigma }_{e}$ is the stress tensor given by

9 $$\begin{aligned} \varvec{\sigma }_{e}=\frac{1}{J}\varvec{F}\cdot 2 \frac{\partial W(\varvec{C})}{\partial \varvec{C}} \cdot \varvec{F}^{T}, \end{aligned}$$

where $W(\varvec{C})$, with $\varvec{C}=\varvec{F}^{T}\varvec{F}$, denotes a strain-energy law (hyperelastic Helmholtz energy functional) dependent on the deformation of the solid. The permeability tensor is given by

10 $$\begin{aligned} \varvec{k}=J^{-1} \varvec{F} \varvec{k}_{0}(\varvec{C}) \varvec{F}^{T} , \end{aligned}$$

where $\varvec{k}_{0}(\varvec{C})$ is the permeability in the reference configuration, which may be chosen to be some (nonlinear) function dependent on the deformation. Examples of deformation dependent permeability tensors for biological tissues can be found in [24, 34, 35]. Details of the derivation of (8) appear in “Appendix 1”.

It is important to recognize that $\nabla ( \cdot )= \partial /\partial \varvec{x} (\cdot )$ denotes the partial derivative with respect to the *deformed* configuration. We will use $\nabla$ to denote the spatial gradient in $\varOmega (t)$ rather than the more explicit $\nabla _{\varvec{x}=\varvec{\chi }(\varvec{X},t)}$. The latter more clearly indicates the dependency of the gradient operator on the deformation $\varvec{\chi }(\varvec{X},t)$ and highlights the inherent nonlinearity that arises due to the fact that the deformation $\varvec{\chi }(\varvec{X},t)$ is one of the unknowns. Similarly the deformed domain $\varOmega (t)$ in which equations (8) pertain, is a function of the deformation map $\varvec{\chi }$, and therefore incorporates another important nonlinearity.

## The stabilized finite element method

We extend the method of [7] from the linear, small deformation poroelastic case to finite-strain poroelasticity. For ease of presentation, we will assume all Dirichlet boundary conditions are homogeneous, ie., $\varvec{u}_{D} = {\varvec{0}}, {q_{D}} = 0, p_{D}=0$.

### Weak formulation

We respectively define the following spaces for the deformed location, fluid flux and pressure,

$$\begin{aligned} \mathbf {W}^{E}(\varOmega (t))= & {} \{ \varvec{v}\in (H^{1} (\varOmega (t)))^d :\varvec{v}= \mathbf{0} \;\text{ on } \;\varGamma _{d}(t) \}, \\ \mathbf {W}^{D}(\varOmega (t))= & {} \{ \varvec{w}\in H_{div}(\varOmega (t)):\varvec{w}\cdot \varvec{n}= 0 \;\text{ on } \;\varGamma _{f}(t) \}, \\ \mathcal {L}(\varOmega (t))= & {} \left\{ \begin{array}{l l} L^{2}(\varOmega (t)) &{}\; \text {if} \; \varGamma _{n}(t) \cup \varGamma _{p}(t) \ne \emptyset \\ L^{2}_{0}(\varOmega (t)) &{}\; \text {if} \;\varGamma _{n}(t) \cup \varGamma _{p}(t) = \emptyset , \end{array} \right\} , \end{aligned}$$

where $$L^{2}_{0}(\varOmega (t)) = \left\{ q \in L^{2}(\varOmega (t)) : \int _{\varOmega (t)} q\;\text{ d }\varOmega (t)=0\right\} $$.

The continuous weak problem is: Find $\varvec{\chi }(\varvec{X},t) \in \mathbf {W}^{E}(\varOmega (0))$, $\varvec{z}(\varvec{x},t) \in \mathbf {W}^{D}(\varOmega (t))$ and $p(\varvec{x},t) \in \mathcal {L}(\varOmega (t))$ for any time $t\in [0,T]$ such that

$$\begin{aligned}&\int _{\varOmega (t)} \left[ \varvec{\sigma }_{e} : \nabla ^{S} \varvec{v}- p\nabla \cdot \varvec{v}\right] \; \mathrm{d}\varOmega (t)\\&\quad = \int _{\varOmega (t)} \rho \varvec{f} \cdot \varvec{v}\; \mathrm{d}\varOmega (t) + \int _{\varGamma _{n}(t)} \varvec{t}_{N} \cdot \varvec{v}\; \mathrm{d}\varGamma _{n}(t) \; \\&\qquad \forall \varvec{v}\in \mathbf {W}^{E}(\varOmega (t)), \\&\int _{\varOmega (t)} \left[ \varvec{k}^{-1}\varvec{z}\cdot \varvec{w}- p\nabla \cdot \varvec{w}\right] \; \mathrm{d}\varOmega (t) \\&\quad =\int _{\varOmega (t)} \rho ^{f} \varvec{f} \cdot \varvec{w}\; \mathrm{d}\varOmega (t) \; \quad \forall \varvec{w}\in \mathbf {W}^{D}(\varOmega (t)), \\ \end{aligned}$$

11 $$\begin{aligned}&\int _{\varOmega (t)} \left[ q\nabla \cdot {\varvec{\chi }_{t}}+ q\nabla \cdot \varvec{z}\right] \; \mathrm{d}\varOmega (t) \nonumber \\&\quad = \int _{\varOmega (t)} g q\; \mathrm{d}\varOmega (t) \; \quad \forall q\in \mathcal {L}(\varOmega (t)). \end{aligned}$$

Here $\nabla ^{S} \varvec{v}=\frac{1}{2}\left( \nabla \varvec{v} + (\nabla \varvec{v})^{T} \right)$ for some vector $\varvec{v}$.

### The fully discrete model

Let $\mathcal {T}^{h}$ be a quasi-uniform partition of $\varOmega (t)$ into non-overlapping elements *K*, where *h* denotes the size of the largest element in $\mathcal {T}^{h}$. We then define the following finite element spaces,

$$\begin{aligned} \mathbf {W}^E_h(\varOmega (t))= & {} \left\{ \varvec{v}_h\in C^{0}(\varOmega (t)): \varvec{v}_h|_{K} \in P_{1}(K) \ \forall K \in \mathcal {T}^{h}, \varvec{v}_h= 0 \; \text{ on } \;\varGamma _{d}(t) \right\} , \\ \mathbf {W}^D_h(\varOmega (t))= & {} \left\{ \varvec{w}_{h}\in C^{0}(\varOmega (t)): \varvec{w}_{h}|_{K} \in P_{1}(K) \ \forall K \in \mathcal {T}^{h}, \varvec{w}_{h}\cdot \varvec{n}= 0 \; \text{ on } \;\varGamma _{f}(t) \right\} , \\ Q_h(\varOmega (t))= & {} \left\{ \begin{array}{l l} \left\{ {q}_{h}: {q}_{h}|_{K} \in P_{0}(K) \ \forall K \in \mathcal {T}^{h} \right\} &{} \; \text {if }\varGamma _{n}(t) \cup \varGamma _{p}(t) \ne \emptyset \\ \left\{ {q}_{h}: {q}_{h}|_{K} \in P_{0}(K), \int _{\varOmega (t)} {q}_{h}=0 \ \forall K \in \mathcal {T}^{h}\right\} &{} \; \text {if } \varGamma _{n}(t) \cup \varGamma _{p}(t)=\emptyset \end{array} \right. , \end{aligned}$$

where $P_{0}(K)$ and $P_{1}(K)$ are the spaces of constant and linear polynomials on *K* respectively. We define the combined solution space $\mathcal {U}_h(t) = \mathbf {W}_h^{E}(\varOmega (0))\times \mathbf {W}_h^{D}(\varOmega (t))\times Q_h(\varOmega (t))$.

The discretization in time is given by partitioning [0, *T*] into *N* evenly spaced non-overlapping regions $(t_{n-1}, t_n]$, $n=1,2,\dots , N$ , where $t_n-t_{n-1} = \varDelta t$. For any sufficiently smooth function *v*(*t*, *x*) we define $v^n(x) = v(t_n,x)$ and the discrete time derivative by $v_{\varDelta t}^{n} := \frac{v^{n}-v^{n-1}}{\varDelta t}$.

The fully discrete weak problem is: For $n = 1, \dots , N$, find $\varvec{\chi }_h^{n} \in \mathbf {W}_h^{E}(\varOmega (0))$, $\varvec{z}_{h}^{n} \in \mathbf {W}_h^{D}(\varOmega (t_n))$ and $p_h^{n} \in Q_h(\varOmega (t_n))$ such that

12 $$\begin{aligned}&\int _{\varOmega (t_n)} \left[ \varvec{\sigma }_{e,h}^{n} : \nabla ^{S} \varvec{v}_h- p_h^{n} \nabla \cdot \varvec{v}_h\right] \; \mathrm{d}\varOmega (t_n) \\&\quad = \int _{\varOmega (t_n)} \rho \varvec{f}^{n} \cdot \varvec{v}_h\; \mathrm{d}\varOmega (t_n) \nonumber \\&\qquad + \int _{\varGamma _{n}(t_n)} \varvec{t}_{N}^{n} \cdot \varvec{v}_h\; \mathrm{d}\varGamma _{n}(t_n) \; \forall \varvec{v}_h\in \mathbf {W}_h^{E}(\varOmega (t_n)), \nonumber \\&\int _{\varOmega (t_n)} \left[ \varvec{k}^{-1}\varvec{z}_{h}^{n} \cdot \varvec{w}_{h}- p_h^{n} \nabla \cdot \varvec{w}_{h}\right] \; \mathrm{d}\varOmega (t_n) \nonumber \\&\quad = \int _{\varOmega (t_n)} \rho ^{f} \varvec{f}^{n} \cdot \varvec{w}_{h}\; \mathrm{d}\varOmega (t_n) \; \forall \varvec{w}_{h}\in \mathbf {W}_h^{D}(\varOmega (t_n)), \nonumber \\&\int _{\varOmega (t_n)} \left[ {q}_{h}\nabla \cdot {\varvec{\chi }^n_{h,\varDelta t}}+ {q}_{h}\nabla \cdot \varvec{z}_{h}^{n} \right] \; \mathrm{d}\varOmega (t_n) + J({{p}^{n}_{h,\varDelta t}},{q}_{h}) \nonumber \\&\quad = \int _{\varOmega (t_n)} g^{n} {q}_{h}\; \mathrm{d}\varOmega (t_n) \; \forall {q}_{h}\in Q_h(\varOmega (t_n)).\nonumber \end{aligned}$$

The stabilization term is given by

$$\begin{aligned} J(p,q)=\varUpsilon \sum _{K \in \mathcal {T}^{h}} \int _{\partial K \backslash \partial \varOmega (t_{n})} h_{\partial K} \llbracket p \rrbracket \llbracket q \rrbracket \; {d}s, \end{aligned}$$

where $\varUpsilon$ is a stabilization parameter that is independent of *h* and $\varDelta t$. Here $h_{\partial K}$ denotes the size (diameter) of an element edge in 2D or face in 3D, and $\llbracket \cdot \rrbracket$ is the jump across an edge or face (taken on the interior edges only). The stabilization term has been introduced here to add stability and ensure a well-posed fully-discrete model. It has been shown that the convergence is insensitive to $\varUpsilon$, e.g. see in [7, 12, 27] .

### Solution via quasi-Newton iteration at $t_n, n=1, \dots , N$.

Let ${\mathfrak {u}_{h}^n}=\{ \varvec{\chi }_h^n, \varvec{z}_{h}^{n}, p_{h}^{n}\} \in \mathcal {U}_h(t_n)$ denote the solution vector at a particular time step, ${\delta \mathfrak {u}_{h}}=\{ \delta \varvec{v}, \delta \varvec{z}, \delta p\}$ denote the solution increment vector, and ${\mathfrak {v}_{h}}=\{ \varvec{v}_h, \varvec{w}_{h}, {q}_{h}\} \in \mathcal {V}_h(t)$ where $\mathcal {V}_h(t) = \mathbf {W}_h^{E}(\varOmega (t))\times \mathbf {W}_h^{D}(\varOmega (t))\times Q_h(\varOmega (t))$. The nonlinear system of Eq. (12) can be recast in the form: Find ${\mathfrak {u}_{h}^n}\in \mathcal {U}_h(t_n)$ such that

13 $$\begin{aligned} G^n({\mathfrak {u}_{h}^n},{\mathfrak {v}_{h}})=0 \; \forall {\mathfrak {v}_{h}}\in \mathcal {V}_h(t_n), \end{aligned}$$

where

14 $$\begin{aligned} \begin{aligned} G^n({\mathfrak {u}_{h}^n},{\mathfrak {v}_{h}})&= \int _{\varOmega (t_n)} \left[ \varvec{\sigma }^{n}_{e,h} : \nabla ^{S} \varvec{v}_h- p_{h}^{n}\nabla \cdot \varvec{v}_h+ \varvec{k}^{-1}\varvec{z}_{h}^{n}\cdot \varvec{w}_{h}\right. \\&\quad \left. - p_{h}^{n}\nabla \cdot \varvec{w}_{h}+\, {q}_{h}\nabla \cdot ( {{{\varvec{v}}^{n}_{\varDelta t,h}}} + \varvec{z}_{h}^{n})\right. \\&\quad \left. - \,\rho \varvec{f}^{n} \cdot \varvec{v}_h+ \rho ^{f} \varvec{f}^{n} \cdot \varvec{w}_{h}+ g {q}_{h}\right] \; \mathrm{d}\varOmega (t_n) \\&\quad -\,\int _{\varGamma _{n}(t_n)} \varvec{t}_{N}^{n} \cdot \varvec{v}_h\; \mathrm{d}\varGamma _{n}(t_n).\\ \end{aligned} \end{aligned}$$

Given an approximate solution $\overline{\mathfrak {u}}_{h}^n$, we approximate (13) by

$$\begin{aligned} G^n(\overline{\mathfrak {u}}_{h}^n,{\mathfrak {v}_{h}}) + DG^n(\overline{\mathfrak {u}}_{h}^n,{\mathfrak {v}_{h}})[ {\delta \mathfrak {u}_{h}}] = 0 \; \forall {\mathfrak {v}_{h}}\in \mathcal {V}_h(t_n), \end{aligned}$$

and solve

15 $$\begin{aligned} DG^n(\overline{\mathfrak {u}}_{h}^n,{\mathfrak {v}_{h}})[{\delta \mathfrak {u}_{h}}]= -G(\overline{\mathfrak {u}}_{h}^n,{\mathfrak {v}_{h}}) \; \forall {\mathfrak {v}_{h}}\in \mathcal {V}_h(t_n), \end{aligned}$$

for the Newton step ${\delta \mathfrak {u}_{h}}$, where *DG* is the directional derivative of *G*, at $\overline{\mathfrak {u}}_{h}^n$, in the direction ${\delta \mathfrak {u}_{h}}$.

#### Approximation of $DG^n$.

In biphasic tissue problems, it is common to approximate directional derivative of *G* by assuming the nonlinear elasticity term is the dominant nonlinearity and ignoring the other nonlinearities [50, 54]. Let

16 $$\begin{aligned} {E}^n((\varvec{\chi }_h^n,p_{h}^{n}) ,\varvec{v}_h)= \int _{\varOmega (t_n)} \left[ \varvec{\sigma }^{n}_{e,h}: \nabla ^{S} \varvec{v}_h- p_{h}^{n}\nabla \cdot \varvec{v}_h\right] \; \mathrm{d}\varOmega (t_n). \end{aligned}$$

For Newton’s method we require the directional derivative of ${E}^n((\varvec{\chi }_h^n,p_{h}^{n}),\varvec{v}_h)$ at a particular trial solution $(\overline{\varvec{\chi }_h^n},\overline{p_{h}^{n}})$ in the direction $\delta \varvec{\chi }_{h}$, given by (see [57, section 3.5.3])

17 $$\begin{aligned} \begin{aligned}&D{E}^n( (\overline{\varvec{\chi }_h^n}, \overline{p_{h}^{n}}) ,\varvec{v}_h)[\delta \varvec{\chi }_{h}] \\&\quad =\int _{\overline{\varOmega }(t_n)} \left[ \nabla ^{S} \varvec{v}_h: \overline{\varvec{\Theta }^{n}_{h}} : \nabla ^{S} \delta \varvec{\chi }_{h}\right. \\&\qquad \left. +\, \overline{\varvec{\sigma }^{n}_{e,h}} : \left( (\nabla \delta \varvec{v})^{T} \cdot \nabla \varvec{v}_h\right) \right] \; \mathrm{d}\varOmega (t_n), \end{aligned} \end{aligned}$$

where $\overline{\varvec{\Theta }^{n}_{h}}$ is a fourth-order tensor and $\overline{\varvec{\sigma }^{n}_{e,h}}$ is the effective (elastic) stress tensor, both evaluated at a trial solution $\overline{\varvec{\chi }_h^n}$. Further, any variable with a bar above it will correspond to it being evaluated at a trial solution. The fourth-order spatial tangent modulus tensor $\varvec{\Theta }$ is described in “Appendix 2”. For a detailed explanation and derivation see [9, 57]. The approximate linearization of the nonlinear problem is thus given by

18 $$\begin{aligned} \begin{aligned}&DG^n(\overline{\mathfrak {u}}_{h}^n, {\mathfrak {v}_{h}}) [{\delta \mathfrak {u}_{h}}] \approx \int _{\overline{\varOmega }(t_n)} \left[ \nabla ^{S} \varvec{v}_h: \overline{{\varvec{\Theta }}^{n}_{h}} : \nabla ^{S} \delta \varvec{\chi }_{h}\right. \\&\quad \left. +\, \overline{\varvec{\sigma }_{e,h}} : \left( (\nabla \delta \varvec{\chi }_{h})^{T} \cdot \nabla \varvec{v}_h\right) - \delta p_{h}\nabla \cdot \varvec{v}_h\right. \\&\quad \left. +\, \bar{\varvec{k}}^{-1}\delta \varvec{z}_{h}\cdot \varvec{w}_{h}- \delta p_{h}\nabla \cdot \varvec{w}_{h}\right. \\&\quad \left. +\, {q}_{h}\nabla \cdot \left( \frac{\delta \varvec{\chi }_{h}}{\varDelta t } + \delta \varvec{z}_{h}\right) \right] \; \mathrm{d}\varOmega (t_n), \end{aligned} \end{aligned}$$

Using (14), (18) and Eq. (15) the Newton solve becomes: Find $\delta \varvec{\chi }_{h}\in \mathbf {W}_h^{E}(\varOmega (0))$, $\delta \varvec{z}_{h}\in \mathbf {W}_h^{D}(\varOmega (t_n))$ and $\delta p_{h}\in Q_h(\varOmega (t_n))$ such that

19 $$\begin{aligned}&\int _{\overline{\varOmega }(t_n)} \left[ \nabla ^{S} \varvec{v}_h: \overline{\varvec{\Theta }^{n}_{h}} : \nabla ^{S} \delta \varvec{\chi }_{h}+ \overline{\varvec{\sigma }^{n}_{e,h}}: \left( (\nabla \delta \varvec{\chi }_{h})^{T} \cdot \nabla \varvec{v}_h\right) \right. \nonumber \\&\qquad \left. - \,{\delta p_{h}} \nabla \cdot \varvec{v}_h\right] \; \mathrm{d}\overline{\varOmega }(t_n) \\&\quad = \int _{\overline{\varOmega }(t_n)} \left[ \overline{\varvec{\sigma }^{n}_{e,h}}: \nabla ^{S} \varvec{v}_h- \overline{p_{h}^{n}} \nabla \cdot \varvec{v}_h- \overline{\rho } \varvec{f}^{n} \cdot \varvec{v}_h\right] \; \mathrm{d}\overline{\varOmega }(t_n) \nonumber \\&\qquad - \int _{\overline{\varGamma }_{n}(t_n)} \varvec{t}_{N}^{n} \cdot \varvec{v}_h\; \mathrm{d}\overline{\varGamma }_{n}(t_n) \quad \forall \varvec{v}_h\in \mathbf {W}_h^{E}(\varOmega (t_n)), \nonumber \\&\int _{\overline{\varOmega }(t_n)} \left[ \bar{\varvec{k}}^{-1}\delta \varvec{z}_{h}\cdot \varvec{w}_{h}- \delta p_{h}\nabla \cdot \varvec{w}_{h}\right] \; \mathrm{d}\overline{\varOmega }(t_n) \nonumber \\&\quad = \int _{\overline{\varOmega }(t_n)} \left[ \bar{\varvec{k}}^{-1}\overline{\varvec{z}_{h}^{n}} \cdot \varvec{w}_{h}- \overline{p_{h}^{n}} \cdot \nabla \varvec{w}_{h}- \overline{\rho ^{f}} \varvec{f}^{n} \cdot \varvec{w}_{h}\right] \; \mathrm{d}\overline{\varOmega }(t_n) \;\;\nonumber \\&\qquad \forall \varvec{w}_{h}\in \mathbf {W}_h^{D}(\varOmega (t_n)), \nonumber \\&\int _{\overline{\varOmega }(t_n)} \left[ {q}_{h}\nabla \cdot \left( \frac{\delta \varvec{\chi }_{h}}{\varDelta t } + \delta \varvec{z}_{h}\right) \right] \;\mathrm{d}\overline{\varOmega }(t_n) + J \left( \frac{ \delta p_{h}}{\varDelta t},{q}_{h}\right) \nonumber \\&\quad = \int _{\overline{\varOmega }(t_n)} \left[ {q}_{h}\nabla \cdot ( \overline{{\varvec{\chi }_{\varDelta t,h}}} + \overline{\varvec{z}_{h}} ) - g {q}_{h}\right] \; \mathrm{d}\overline{\varOmega }(t_n) \;\nonumber \\&\qquad + J\left( \overline{{{p}_{h,\varDelta t}}},{q}_{h}\right) \quad \forall {q}_{h}\in Q_h(\varOmega (t_n)).\nonumber \end{aligned}$$

## Implementation details

### Matrix assembly for the Newton iteration

Let $\varvec{\phi }_{k}$ denote a vector-valued linear basis function for the (P1)$^d$ space, and

$$\begin{aligned} \varvec{\chi }^{n}_{i}= & {} \sum _{k=1}^{n_{u}}\varvec{\chi }^{n}_{i,k}\varvec{\phi }_{k} \in \mathbf {W}_h^{E}(\varOmega (0)), \\ \varvec{z}^{n}_{i}= & {} \sum _{k=1}^{n_{z}}\varvec{z}^{n}_{i,k}\varvec{\phi }_{k} \in \mathbf {W}_h^{D}(\varOmega (t_n)). \end{aligned}$$

Similarly let ${\psi }_{i}$ denote a basis function for the space P0, hence

$$\begin{aligned} \varvec{p}^{n}_{i}= \sum _{k=1}^{n_{p}}{p}^{n}_{i,k}{\psi }_{k} \in Q_h(\varOmega (t_n)). \end{aligned}$$

Now let $\mathfrak {u}_{i}^{n}:=( \varvec{\chi }_{i}^{n}, \varvec{z}_{i}^{n}, p_{i}^{n} ) \in \mathbb {R}^{n_u+n_z+n_p}$ denote the fully discrete solution at the *i*th step within the Newton method at time $t_{n}$. The Newton algorithm at a particular time step *n*, is given in Algorithm 1.
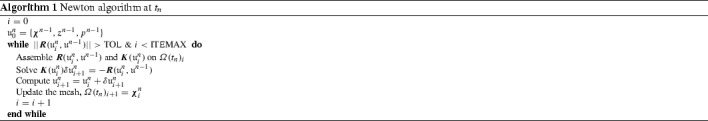


At each Newton iteration we are required to solve the linear system

20 $$\begin{aligned} \varvec{K}(\mathfrak {u}_{i}^{n}) \delta \mathfrak {u}_{i+1}^{n} = - \varvec{R}(\mathfrak {u}_{i}^{n},\mathfrak {u}^{n-1}). \end{aligned}$$

This system can be expanded as

21 $$\begin{aligned} \begin{bmatrix} \varvec{K}^{e}&\quad 0&\quad \varvec{B}^{T} \\ 0&\quad \varvec{M}&\quad \varvec{B}^{T} \\ -\varvec{B}&\quad -\varDelta t \varvec{B}&\quad \varvec{J} \end{bmatrix} \begin{bmatrix} \delta \varvec{\chi }^{n}_{i+1} \\ \delta \varvec{z}^{n}_{i+1} \\ \delta {p}^{n}_{i+1} \end{bmatrix}= - \begin{bmatrix} \varvec{r}_{1}(\varvec{\chi }^{n}_{i},{p}^{n}_{i}) \\ \varvec{r}_{2}(\varvec{\chi }^{n}_{i},\varvec{z}^{n}_{i},{p}^{n}_{i}) \\ \varvec{r}_{3}(\varvec{\chi }^{n}_{i},\varvec{\chi }^{n-1},\varvec{z}^{n}_{i},{p}^{n}_{i}) \end{bmatrix}, \end{aligned}$$

where the elements in the matrices in (21) are given by

$$\begin{aligned}&\varvec{k}^{e}_{kl} = \int _{{\varOmega (t_n)_{i} }} \left[ \varvec{E}^{T}_{k}\varvec{D}(\varvec{\chi }^{n}_{i})\varvec{E}_{l} + (\nabla \varvec{\phi }_{k})^{T}\varvec{\sigma }_{e}(\varvec{\chi }^{n}_{i})\nabla \varvec{\phi }_{l} \right] \; \mathrm{d}\varOmega (t_n)_{i}, \\&\varvec{m}_{kl} = \int _{{\varOmega (t_n)_{i} }} \varvec{k}^{-1}(\varvec{\chi }^{n}_{i}) \varvec{\phi }_{k} \cdot \varvec{\phi }_{l} \; \mathrm{d}\varOmega (t_n)_{i}, \\&\varvec{b}_{kl} = -\int _{{\varOmega (t_n)_{i} }} {\psi }_{k} \nabla \cdot \varvec{\phi }_{l} \; \mathrm{d}\varOmega (t_n)_{i}, \\&\varvec{j}_{kl} = \varUpsilon \sum _{K \in \mathcal {T}^{h}_{i}} \int _{\partial K \backslash \partial {\varOmega (t_n)_{i} }} h_{\partial K} \llbracket {\psi }_{k}\rrbracket \llbracket {\psi }_{l}\rrbracket \;\mathrm{d}s. \\&\varvec{r}_{1i} = \int _{{\varOmega (t_n)_{i}}} \left[ \left( \varvec{\sigma }_{e}(\varvec{\chi }^{n}_{i})-p^{n}_{i} \varvec{I} \right) : \nabla \varvec{\phi }_{i} - {\rho }(\varvec{\chi }^{n}_{i}) \varvec{\phi }_{i}\cdot \varvec{f} \right] \; \mathrm{d}\varOmega (t_n)_{i} \\&\qquad \qquad - \int _{\varGamma _{n}(t_n)_{i}} \varvec{\phi }_{i} \cdot \varvec{t}_{N}(\varvec{\chi }^{n}_{i}) \; \mathrm{d}\varGamma _{n}(t_n)_{i}, \\&\varvec{r}_{2i} = \int _{{\varOmega (t_n)_{i}}} \left[ \varvec{k}^{-1}(\varvec{\chi }^{n}_{i}) \varvec{\phi }_{i} \cdot \varvec{z}^{n}_{i} - {p}^{n}_{i} \nabla \cdot \varvec{\phi }_{i} - {\rho ^{f}} \varvec{\phi }_{i}\cdot \varvec{f} \right] \; \mathrm{d}\varOmega (t_n)_{i} , \\&\varvec{r}_{3i} = \int _{{\varOmega (t_n)_{i}}} {\psi }_{i} \left[ \nabla \cdot \left( {\varvec{\chi }^{n}_{i}-\varvec{\chi }^{n-1}} \right) + \varDelta t {\psi }_{i} \nabla \cdot \varvec{z}^{n}_{i} - \varDelta t{\psi }_{i} g \right] \; \mathrm{d}\varOmega (t_n)_{i} \\&\qquad \qquad +\,\varUpsilon \sum _{K \in \mathcal {T}^{h}_{i}} \int _{\partial K \backslash \partial {\varOmega (t_n)_{i} }} h_{\partial K} \llbracket {\psi }_{i}\rrbracket \llbracket {{p}^{n}_{i}-{p}^{n-1}}\rrbracket \; \mathrm{d}s. \end{aligned}$$

The saddle point system given in Eq. (21) can be iteratively solved using similar approaches to those seen in [29, 48], where the action of the inverse of the preconditioner fundamentally requires only the inverse of the linear blocks $K^e$ and *M*. Details of the matrices $\varvec{D}$ and $\varvec{E}$ appear in “Appendix 2”. Note that the matrix equations are integrated in the deformed configuration obtained from the previous Newton step. This update Lagrangian approach overcomes complex linearisation otherwise needed when using a total Lagrangian approach [50]. The Newton iteration was found to be robust with respect to the stabilization parameter. More precisely, in all calculations fewer than four Newton iterations were required independently of the size of the stabilization parameter. Tables 2 and 3 in Sect. 5.1 show the Newton convergence for two choices of the stabilization parameter two orders of magnitude apart for a 3D stress relaxation text problem. In practice, the stabilization parameter was chosen so as to be as small as possible without producing oscillations, unless otherwise stated.

### Stabilization matrix assembly

Let $K \in \mathcal {T}_{h}$ be an element and $\mathcal {D}(K)$ be the pressure degree of freedom associated with element *K*. We define $\mathcal {A}(K)$ to be the set of elements $L \in \mathcal {T}_{h}$ neighboring *K*.
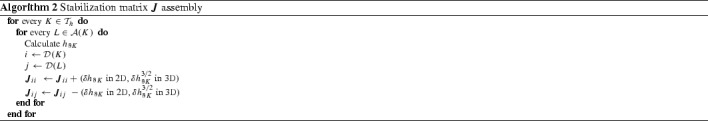


### Fluid-flux boundary condition

When solving the equations for Darcy flow using the Raviart–Thomas element (RT-P0), the fluid-flux boundary condition is enforced naturally by this divergence free element. Unfortunately this is not possible using our proposed P1-P1-P0-stabilized element. However, solving the poroelastic equations (8) using a piecewise linear approximation for the deformation and Raviart–Thomas element for the fluid (P1-RT-P0) does not satisfy the discrete inf-sup condition and can yield spurious pressure oscillations, see [46, 47] for details.

To enforce the flux boundary condition $\mathbf {z}\cdot \varvec{n}= q_{D}$ along the boundary $\varGamma _{f}(t)$ we introduce a Lagrange multiplier $\varLambda _h$, where $\varLambda _h \in W_h^f(t)$, the discrete space of piecewise constant functions defined on all element surfaces with non-zero intersection with $\varGamma _f(t)$. The resulting modified continuous weak-form is

22 $$\begin{aligned}&G( (\varvec{\chi }_h,\varvec{z}_{h},p_h), (\varvec{v}_h,\varvec{w}_{h},{q}_{h}) ) \nonumber \\&\quad + (\varLambda _h,\varvec{w}_{h}\cdot \varvec{n})_{\varGamma _{f}} =0, \quad \forall (\varvec{v}_h,\varvec{w}_{h},{q}_{h}) \in \mathcal {V}_h(t),\end{aligned}$$

23 $$\begin{aligned}&\qquad (\varvec{z}_{h}\cdot \varvec{n},\varvec{l})_{\varGamma _{f}} =q_{D}, \quad \forall \varvec{l}\in W_h^f(t). \end{aligned}$$

The discretization and implementation of this additional constraint is straightforward and results in a discrete system with additional degrees of freedom for every node on $\varGamma _{f}$. The terms $(\varLambda _h,~\varvec{w}~\cdot ~\varvec{n})_{\varGamma _{f}}$ and $(\varvec{z}~\cdot ~\varvec{n},~\varvec{l})_{\varGamma _{f}}$ are nonlinear since the normal is a function of the (nonlinear) displacement. Note, within all the simulations we have undertaken, we found that treating these terms as linear terms did not prevent the convergence of the Newton algorithm. Alternatively these terms could be linearized as has been described in detail for the traction boundary condition, see [57, section 4.2.5] and [4].

## Numerical results

We present four numerical examples to test the performance of the proposed stabilized finite element method. The first two examples are biological and geomechanical applications respectively, and the third is a swelling example that undergoes significant, large deformations. For the implementation we used the C++ library libmesh [33], and the multi-frontal direct solver mumps [2] to solve the resulting linear systems. For the strain energy law we chose a Neo-Hookean law taken from [57, eqn. (3.119)], with the penalty term chosen such that $0\le \phi <1$, namely

24 $$\begin{aligned} W(\varvec{C})=\frac{\mu }{2}(\text{ tr }(\varvec{C})-3) +\frac{\lambda }{4}(J^{2}-1)-\left( \mu +\frac{\lambda }{2}\right) \text{ ln }(J-1+\phi _{0}). \end{aligned}$$

For further discussion of strain energy laws for porelasticity we refer to [14] and [52]. The material parameters $\mu$ and $\lambda$ in (24) can be related to the Young’s modulus *E* and the Poisson ratio $\nu$ by $\mu =E/(2(1+\nu ))$ and $\lambda =(E\nu )/((1+\nu )(1-2\nu ))$. Details of the effective stress tensor and fourth-order spatial tangent modulus for this particular law can be found in “Appendix 2”. For the permeability law we chose

25 $$\begin{aligned} \varvec{k}_{0}(\varvec{C})=k_{0}\varvec{I}. \end{aligned}$$

### 3D unconfined compression problem

**Fig. 2:**
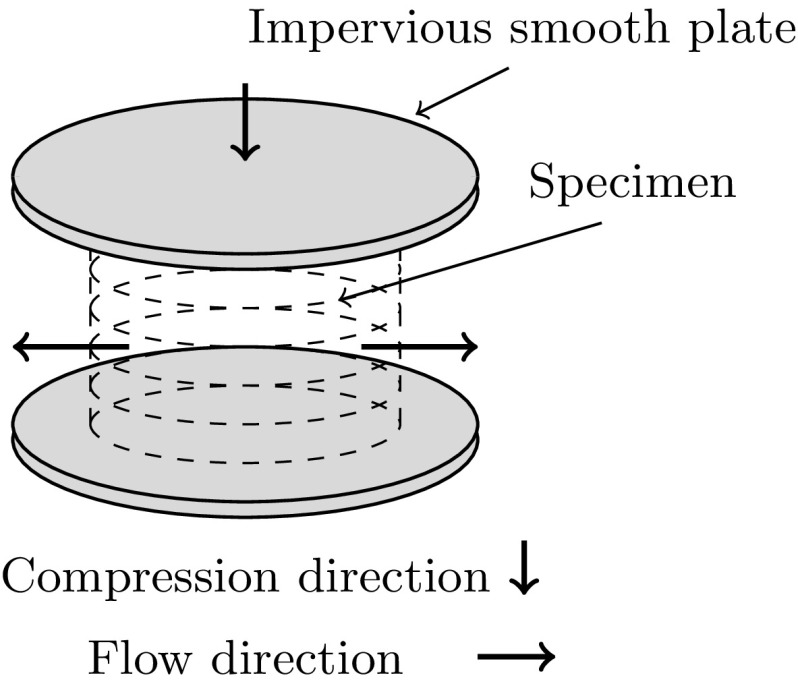
The 3D unconfined compression problem

This first example tests the correctness of the implementation by comparing the numerical solution of the finite-strain system of equations to one of the very few available analytical solutions for poroelastic problems, all-be-it for small deformations. Similar unconfined compression problems have been used to test other large deformation poroelastic software such as FEBio [40]. A cylinder of poroelastic materials is subjected to a prescribed displacement in the axial direction. The material is allowed to relax in the radial direction by constraining the fluid pressure to be zero at the outer radial surface and assuming the outer radial boundary is permeable and free-draining. The upper and lower fluid boundaries are assumed to be impermeable and frictionless. The original experiment involved a specimen of articular cartilage being compressed via impervious smooth plates as shown in Fig. 2. Both the radius and height of the cylinder are 1 mm, whereas the axial compression is 0.01 mm, hence the axial compression is small compared to the size of the height of the cylinder. Large deformation effects are therefore expected to be negligible. The parameters used for the simulation can be found in Table 1. All computations were performed using 3080 tetrahedral elements.

**Table 1:** Parameters used for the 3D unconfined compression test problem

Parameter	Description	Value
$\phi _{0}$	Initial fluid volume fraction	0.9
$k_{0}$	Dynamic permeability	$10^{-3} \; \text{ m }^{3}\,\text{ s }\,\text{ kg }^{-1}$
$\nu$	Poisson’s ratio	0.15
*E*	Young’s modulus	1000 $\text{ kg }\,\text{ m }^{-1}\,\text{ s }^{-2}$
$\varDelta t$	Time step used in the simulation	$4\,\text{ s }$
*T*	Final time of the simulation	$1000\,\text{ s }$
$$\varUpsilon $$	Stabilization parameter	$$ 10^{-3}$$

A closed-form axisymmetric solution for small strains [3] is

26 $$\begin{aligned} \frac{u}{a}(a,t)=\epsilon _{0} \left[ \nu + (1-2\nu )(1-\nu ) \sum ^{\infty }_{n=1} \frac{\exp \left( -\alpha _n^{2} \frac{Mkt}{a^{2}}\right) }{\alpha _{n}^{2}(1-\nu )^{2}-(1-\nu )} \right] , \end{aligned}$$

where *u* is the radial displacement, $\epsilon _{0}$ is the amplitude of the applied axial strain and *a* is the radius of the cylinder. Here $\alpha _n$ are the solutions to the characteristic equation, given by $J_{1}(x)-(1-\nu )xJ_{0}(x)/(1-2\nu )=0,$ where $J_{0}$ and $J_{1}$ are Bessel functions. The characteristic time of diffusion $t_{g}$ is given by $t_{g}= a^{2}/M k$, where $M=\lambda + 2\mu$ is the P-wave modulus of the elastic solid skeleton, *k* is the permeability.

**Fig. 3:**
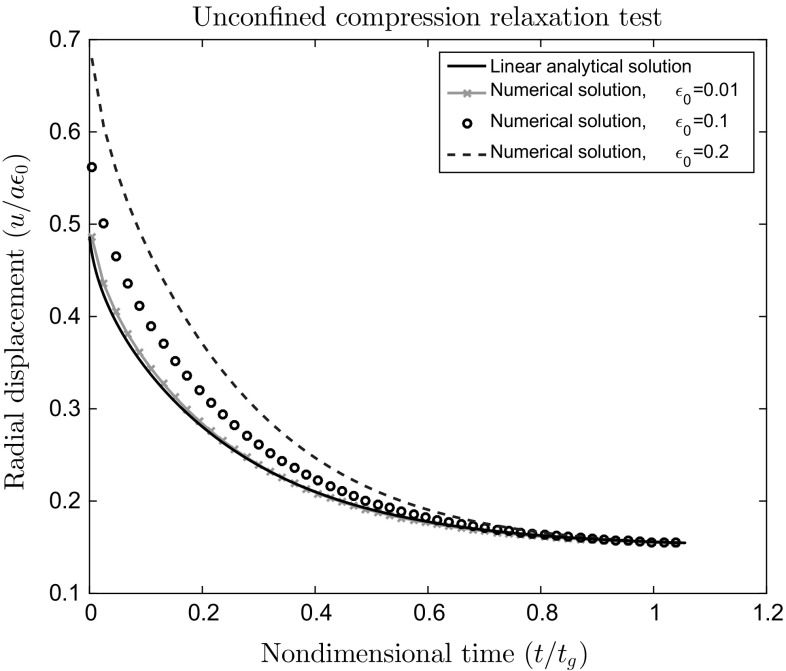
The 3D unconfined compression problem. Normalized radial displacement versus normalized time for vertical normalized displacements $\epsilon _{0}=0.01, 0.1, 0.2$ compared to the analytical, infinitesimal strain solution. All computations performed with $\varUpsilon =0.001$

For small axial compression the computed radial displacement shown in Fig. 3 is in good agreement with the analytical solution, indicating that the nonlinear poroelastic model is accurate in the small strain limit. As the axial compression becomes large, the numerical finite strain solution departs from the analytical linear small deformation solution as expected.

**Fig. 4:**
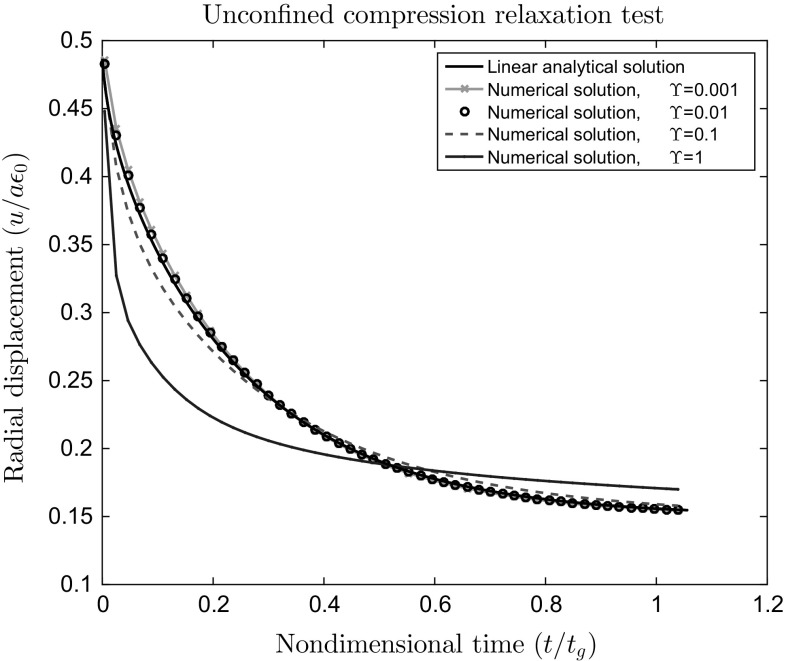
The 3D unconfined compression problem. Normalized radial displacement versus normalized time calculated using stabilized finite element method for $\epsilon _{0}=0.01$ using various values of $\varUpsilon$ and compared to the analytical, infinitesimal strain solution

The effect of the stabilization parameter on the numerical solution is investigated in Fig. 4, and shown to be robost for a broad range of values, since the stabilization parameter can be chosen to be very small in 3D. Without stabilization the Newton method solving the non-linear equations diverged rapidly due to spurious pressure modes present at each Newton step and a solution could not be obtained. Further tests to investigate this type of loss of stability are given in Sect. 5.3.

Tables 2 and 3 illustrate convergence of the Newton iteration for the unconfined compression problem for the first time step (the most demanding due to the initial displacement boundary condition), with $\varUpsilon =10^{-1}$ and $\varUpsilon =10^{-3}$, respectively. The Newton convergence is minimally affected by the decrease in stabilization. The Newton iteration fails to converge if the stabilization parameter is further reduced to $\varUpsilon =10^{-6}$, and as a consequence no solution can be obtained. The linear system contains 8162 degrees of freedom, takes $15.25\,\text{ s }$ to assemble and $1.57\, \text{ s }$ to solve, using one Intel Xenon CPU.

**Table 2:** Convergence of the Newton iteration for the 3D unconfined compression problem with $\varUpsilon =10^{-1}$ at $t=4\,\text{ s }$

Newton iteration	$||\mathfrak {u}_{i}^{n}-\mathfrak {u}^{n}_{i-1}||$	$||\varvec{R}(\mathfrak {u}_{i}^{n},\mathfrak {u}^{n-1})||$
1	0.81	0.023202
2	2.81699e−04	0.011276
3	6.93986e−08	1.34048e−06
4	4.10726e−10	7.64882e−09

**Table 3:** Convergence of the Newton iteration for the 3D unconfined compression problem with $\varUpsilon =10^{-3}$ at $t=4\,\text{ s }$

Newton iteration	$||\mathfrak {u}_{i}^{n}-\mathfrak {u}^{n}_{i-1}||$	$||\varvec{R}(\mathfrak {u}_{i}^{n},\mathfrak {u}^{n-1})||$
1	0.81	0.0235609
2	1.28528e−05	1.99541e−04
3	7.71658e−08	1.49304e−06
4	4.6844e−10	8.17681e−09

### Terzaghi’s problem

Terzaghi’s problem is a common test problem within the geomechanics community that has an analytical solution. It has been used to investigate the origins of non-physical pressure oscillations arising in some finite element solutions near the boundary [43, 54]. The domain consists of a porous column of unit height, bounded at the sides and bottom by rigid and impermeable walls. The top is free to drain ($p_{D}=0$) and has a downward traction force, $p_{0}$, applied to it. The boundary and initial conditions for this 1D problem can be written as

27 $$\begin{aligned} \begin{array}{ccccc} \begin{aligned} t_{N} = -p_{0}, \\ u=0, \\ u=0, \end{aligned}&{} \; \begin{aligned} &{} \\ &{}z=0 \\ &{}z=0, \end{aligned}&{} \; \begin{aligned} &{}p_{D}=0\\ \ &{}\\ &{}p=0 \end{aligned}&{} \; \begin{aligned} \text{ for } \; &{}x=0, \; t>0\\ \text{ for } \; &{}x=1, \; t>0\\ \text{ for } \; &{}x \in [0,1], \; t=0. \end{aligned} \end{array} \end{aligned}$$

The analytical pressure solution, in non-dimensional form is given by

28 $$\begin{aligned} p^{*}(x,t)= & {} \sum _{n}^{\infty }\frac{2}{\pi (n+1/2)} \sin (\pi (n+1/2)x)\nonumber \\&\quad \exp ^{-\pi (n+1/2)(\lambda +2\mu )k t}. \end{aligned}$$

For a detailed explanation and derivation of this solution see [16, section 5.2.2]. We discretized the column using 60 hexahedral elements and solved the problem using both the stabilized low-order finite element method and a higher-order inf-sup stable finite element method with piecewise linear pressure approximation. The material parameters used for the simulation can be found in Table 4.

**Fig. 5:**
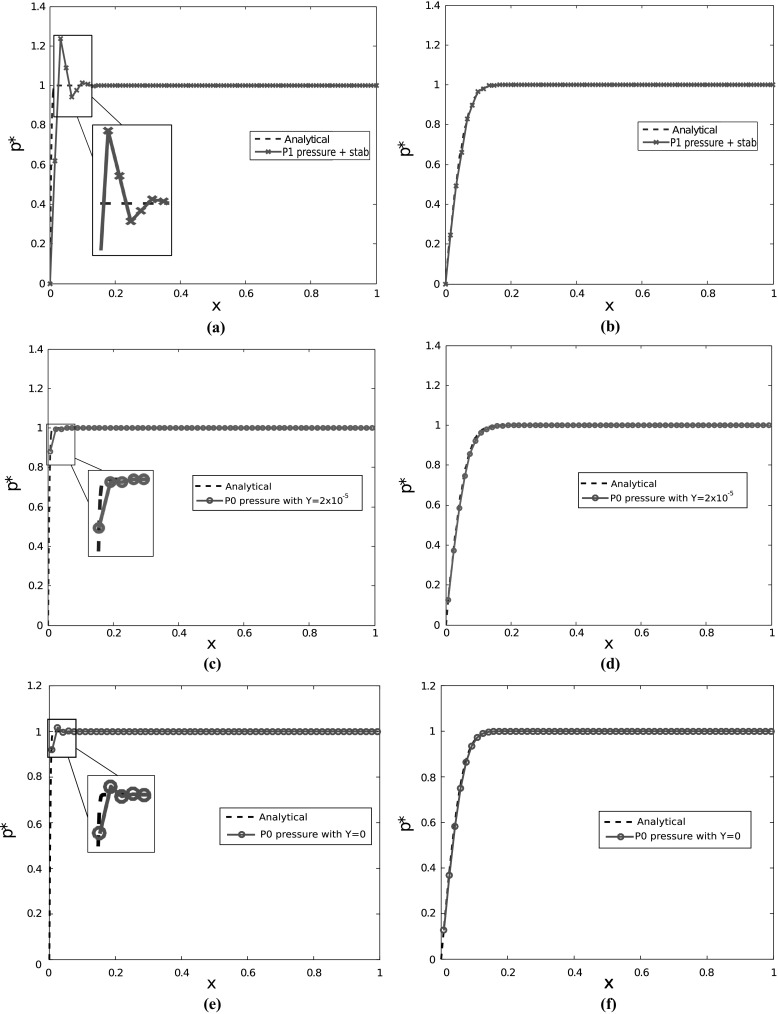
Terzaghi’s problem. **a** Pressure at $t=0.01\,\text{ s }$ using a continuous linear pressure approximation. **b** Pressure at $t=1s$ using a continuous linear pressure approximation. **c** Pressure at $t=0.01\,\text{ s }$ using a discontinuous piecewise constant approximation with $\varUpsilon =2\times 10^{-5}$. **d** Pressure at $t=1\,\text{ s }$ using a discontinuous piecewise constant approximation with $\varUpsilon =2\times 10^{-5}$. **e** Pressure at $t=0.01\,\text{ s }$ using a discontinuous piecewise constant approximation without stabilization. **f** Pressure at $t=1\,\text{ s }$ using a discontinuous piecewise constant approximation without stabilization.

The simulation results of the pressure for the two methods at $t=0.01\,\text{ s }$ and $t=1\,\text{ s }$ are shown in Fig. 5. At $t=0.01\,\text{ s }$ the piecewise linear (continuous) approximation, which is inf-sup stabilized using a Brinkman term [18], fails to approximate the thin boundary layer in the pressure field and suffers from overshooting (Fig. 5a). The stabilized low-order method does not suffer from this problem and accurately captures the pressure field near the boundary (Fig. 5c). At $t=1\,\text{ s }$ the boundary layer has grown and both the piecewise linear pressure approximation (Fig. 5b) and the piecewise constant pressure approximations (Fig. 5d) yield satisfactory results. Note that even when using discontinuous pressure interpolation, pressure oscillations are present without stabilization at $t=0.01\,\text{ s }$, see Fig. 5e. Again this pressure oscillation disappears as the pressure boundary layer grows with time and the lack of inf-sup stability is not obvious from the solution at $t=1\,\text{ s }$, see Fig. 5f.

**Table 4:** Parameters used for Terzaghi’s problem

Parameter	Description	Value
$\phi _{0}$	Initial fluid volume fraction	0.9
$k_{0}$	Dynamic permeability	$10^{-5} \; \text{ m }^{3}\,\text{ s }\,\text{ kg }^{-1}$
$\nu$	Poisson ratio	0.25
*E*	Young’s modulus	100 $\text{ kg }\,\text{ m }^{-1}\,\text{ s }^{-2}$
$\varDelta t$	Time step used in the simulation	$0.01\,\text{ s }$
*T*	Final time of the simulation	$1\,\text{ s }$
$\varUpsilon$	Stabilization parameter	$2\times 10^{-5}$

**Fig. 6:**
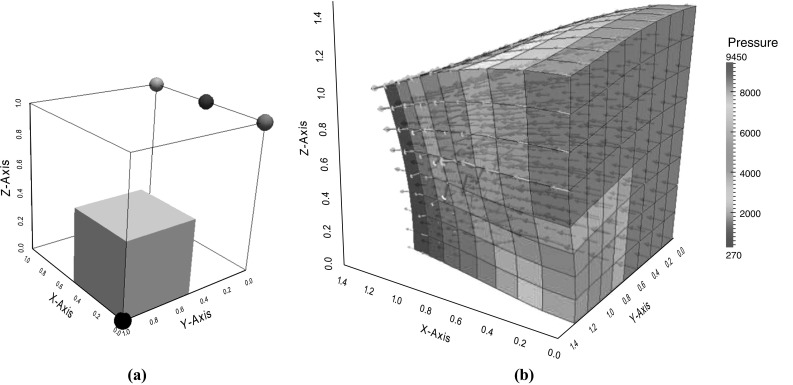
Swelling test. **a** Initial configuration. The *grey cube* represents the region of reduced permeability. The *colored balls* indicate the position of the points used for tracking the pressure and volume changes shown in Fig. 7a and b. **b** The deformed cube after 1 s showing the pressure solution and fluid flux

### Swelling test

Given a unit cube of material, a fluid pressure gradient is imposed between the two opposite faces at $X=0$ and $X=1$. The pressure $p_{D}$ on the inlet face $X = 0$ is increased very rapidly from zero to a limiting value of $10 \text{ kPa }$, i.e., $p_{D} = 10^{4} (1-\text{ exp }(-t^{2}/0.25)) \;\text{ Pa })$. On the outlet face $X = 1$, the pressure is fixed to be zero, $p_{D} =0$. There are no sources of sinks of fluid. A zero flux condition is applied for the fluid velocity on the four other faces ($Y=0,1, \;Z=0,1$). Normal displacements are required to be zero on the planes $X = 0, \ Y = 0$ and $Z = 0$. The permeability of the cube $0< X< 0.5, \ 0.5< Y< 1, \ 0< Z <0.5$, i.e., 1/8 of the volume, is smaller than in the rest of the unit cube by a factor of 500. The computational domain is shown in Fig. 6a, highlighting the region of reduced permeability. The parameters chosen for this test problem are given in Table 5. This problem is similar to the one in [13] and highlights the method’s ability to reliably capture steep gradients in the pressure solution due to rapid changes in material parameters.

**Fig. 7:**
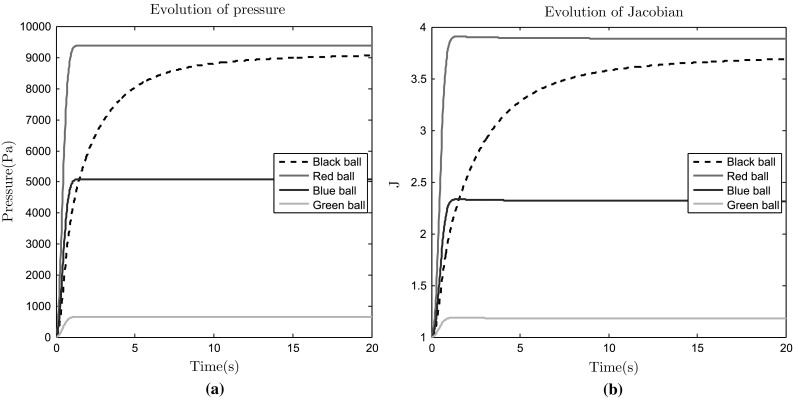
Swelling test. **a** Pressure, *p*, at locations $\varvec{X}=(0,0,1)$ [*red*], $\varvec{X}=(0.5,0,1)$ [*blue*], $\varvec{X}=(1,0,1)$ [*green*] and $\varvec{X}=(1,0,1)$ [*black*]. **b** Volume change, *J* (**b**) at locations $\varvec{X}=(0,0,1)$ [*red*], $\varvec{X}=(0.5,0,1)$ [*blue*], $\varvec{X}=(1,0,1)$ [*green*] and $\varvec{X}=(1,0,1)$ [*black*]. (These locations are shown using the *colored balls* in Fig. 6a)

Fluid enters the region from the inlet face and the material swells like a sponge, undergoing large deformation as shown in Fig. 6b. The evolution of the pressure and the Jacobian at the points at $(0,0,1), \ (0.5,0,1)$ and (1, 0, 1) in the reference configuration are shown in Fig. 7a and b respectively. These positions are indicated by the red, blue and green balls in Fig. 6a. The pressure and volume change at the point (0, 1, 0) (black ball in Fig. 6a) is also shown in Fig. 7a and b. Due to its reduced permeability, this region is much slower to swell and achieve its ultimate equilibrium state and the fluid mainly flows around the region of reduced permeability, see Fig. 6b. The steep pressure gradients at the boundary of the less permeable region seen in Fig. 6b are well approximated by the piecewise constant (discontinuous) pressure space even on this relatively coarse discretization, and the no-flux boundary condition is enforced correctly along the deformed boundary. Continuous pressure spaces would require a much finer discretization in this region.

**Table 5:** Parameters used for the swelling test problem

Parameter	Description	Value
$\phi _{0}$	Initial fluid volume fraction	0.9
$k_{0}$	Dynamic permeability	$10^{-5} \; \text{ m }^{3}\,\text{ s }\,\text{ kg }^{-1}$
$\nu$	Poisson ratio	0.3
*E*	Young’s modulus	8000 $\text{ kg }\,\text{ m }^{-1}\,\text{ s }^{-2}$
$\varDelta t$	Time step used in the simulation	$0.02\,\text{ s }$
*T*	Final time of the simulation	$20\,\text{ s }$
$\varUpsilon$	Stabilization parameter	$10^{-4}$

**Fig. 8:**
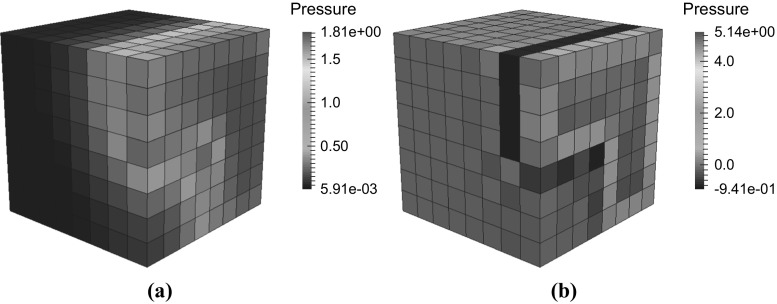
Swelling test problem with non-uniform permeability. Pressure field at $t=0.02\,\text{ s }$ using the stabilized finite element method. **a** $\varUpsilon =10^{-4}$, **b** $\varUpsilon =0$

**Fig. 9:**
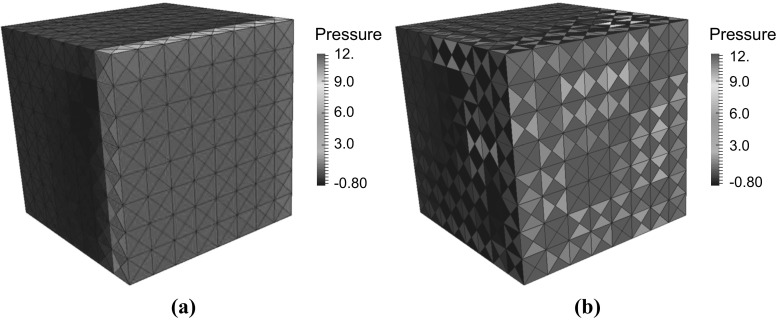
Swelling problem with uniform permeability. Tetrahedral mesh. **a** Pressure field at $t=0.02\,\text{ s }$ using the stabilized finite element method. **a** $\varUpsilon =10^{-4}$, **b** $\varUpsilon =10^{-12}$

Figure 8 shows the pressure solution for this test problem with ($\varUpsilon =10^{-4}$ in Fig. 8a) and without stabilization ($\varUpsilon =0$ in Fig. 8b) at $t=0.02\,\text{ s }$. The computation was performed using 512 hexahedral elements. Note that without any stabilization pressure an instability in the pressure is observed.

To further investigate and demonstrate that a lack of stabilization will result in a loss of inf-sup stability and thus result in a spurious chequer board pressure solution, we run the same swelling test problem, but with a homogeneous permeability permeability set at $k_{0}=10^{-5}$. Furthermore we solve this problem on a 12,288 element tetrahedral mesh, which has a smaller ratio of fluid and displacement nodes to pressure nodes, compared to the previously used hexahedral mesh, thus worsening the inf-sup instability properties [18]. Figure 9 shows the pressure solution with ($\varUpsilon =10^{-4}$ in Fig. 9a) and ($\varUpsilon =\varUpsilon =10^{-12}$ in Fig. 9b) at $t=0.02\,\text{ s }$. Note that without sufficient stabilization the lack of inf-sup stability can clearly be observed in the form of a spurious pressure checkerboard solution. These numerical examples demonstrate that the stabilization scheme is very robust to ensuring inf-sup stability by allowing a large range of stabilization parameters to be used, before spurious pressure oscillations caused by a loss of inf-sup stability are observed. However when wishing to capture pressure boundary layer type solutions, care does need to be taken to ensure that the pressure solution is not overly smoothed. As a practical guide we recommend first choosing a large stabilization parameter and then repeatedly lowering the stabilization parameter by an order of magnitude until the lowest parameter is found that leads to a pressure solution without any oscillations.

## Conclusions

Stabilized low-order methods can offer significant computational advantages over higher order approaches. In particular, one can employ meshes with fewer degrees of freedom, fewer Gauss points, and simpler data structures. The additional stabilization terms can also improve the convergence properties of iterative solvers.

The main contribution of this paper is to extend the local pressure jump stabilization method [12] already applied to three-field linear poroelasticity in [7], to the finite strain case. Thus, the proposed scheme is built on an existing scheme for which rigorous theoretical results addressing the stability and optimal convergence have been proven, and for which numerical experiments have demonstrated its ability to overcome spurious pressure oscillations. Owing to the discontinuous pressure approximation, sharp pressure gradients due to changes in material coefficients or boundary layers can be captured reliably, circumventing the need for severe mesh refinement. The addition of the stabilization term introduces minimal additional computational work, can be assembled locally on each element using standard element information, and leads to a symmetric addition to the original system matrix, thus preserving any existing symmetry. As the numerical examples have demonstrated, the stabilization scheme is robust and leads to high-quality solutions.

## Appendix 1: Model derivation

### Conservation of mass

The mass balance for the solid and fluid phases respectively can be expressed as

29 $$\begin{aligned}&\frac{d}{dt}\int _{\varOmega (t)}(1-\phi )\rho ^{s} \; \mathrm{d}\varOmega (t)\nonumber \\&\quad = \int _{\varOmega (t)} \left( \frac{\partial (1-\phi )\rho ^{s} }{\partial t} + \nabla \cdot ( (1-\phi )\rho ^{s}\varvec{v}^{s}) \right) \; \mathrm{d}\varOmega (t) = 0, \end{aligned}$$

30 $$\begin{aligned}&\frac{d}{dt}\int _{\varOmega (t)}\phi \rho ^{f} \; \mathrm{d}\varOmega (t) \nonumber \\&\quad = \int _{\varOmega (t)} \left( \frac{\partial \phi \rho ^{f} }{\partial t} + \nabla \cdot ( \phi \rho ^{f} \varvec{v}^{f}) \right) \; \mathrm{d}\varOmega (t) = \int _{\varOmega (t)} \rho ^f g \; \mathrm{d}\varOmega (t), \nonumber \\ \end{aligned}$$

where $\varvec{v}^{f}$ is the velocity of the fluid and $\varvec{v}^{s}$ is the velocity of the solid given by

31 $$\begin{aligned} \varvec{v}^{s}(\varvec{x},t)|_{\varvec{x}=\varvec{\chi }(\varvec{X},t)}= \frac{\partial \varvec{\chi }(\varvec{X},t)}{\partial t} \,. \end{aligned}$$

and *g* is a general source or sink term. In differential form,

32 $$\begin{aligned} \frac{\partial (1-\phi )\rho ^{s} }{\partial t} + \nabla \cdot ( (1-\phi )\rho ^{s}\varvec{v}^{s})= & {} 0 \quad \text{ in }\;\varOmega (t), \end{aligned}$$

33 $$\begin{aligned} \frac{\partial (\phi \rho ^{f}) }{\partial t} + \nabla \cdot ( \phi \rho ^{f} \varvec{v}^{f})= & {} \rho ^f g \quad \text{ in }\;\varOmega (t), \end{aligned}$$

or,

34 $$\begin{aligned} \frac{\partial \hat{\rho }^{s} }{\partial t} + \nabla \cdot ( \hat{\rho }^{s}\varvec{v}^{s})= & {} 0 \quad \text{ in }\;\varOmega (t), \end{aligned}$$

35 $$\begin{aligned} \frac{\partial \hat{\rho }^{f} }{\partial t} + \nabla \cdot ( \hat{\rho }^{f} \varvec{v}^{f})= & {} \rho ^f g \quad \text{ in }\;\varOmega (t), \end{aligned}$$

where $$\hat{\rho }^{s}=\rho ^{s}(1-\phi )$$ and $$\hat{\rho }^{f}=\rho ^{f}\phi $$.

First noting that $\rho ^{s}$ and $\rho ^{f}$ are constants and can be factored out and then adding Eqs. (32) and (33), provides the mass balance or continuity equation of the mixture,

36 $$\begin{aligned} \nabla \cdot ( (1-\phi )\varvec{v}^{s}) + \nabla \cdot ( \phi \varvec{v}^{f}) = g \quad \text{ in }\;\varOmega (t). \end{aligned}$$

### Conservation of momentum

For $\alpha =s,f$ the conservation of linear momentum for solid and fluid components is given by

37 $$\begin{aligned} \frac{d}{dt} \int _{\varOmega (t)} \hat{\rho }^{\alpha }\varvec{v}^{\alpha } d\varOmega (t) =\int _{\varOmega (t)} \nabla \cdot \varvec{\sigma }^{\alpha } + \hat{\rho }^{\alpha } {\varvec{f}} + \hat{\varvec{p}}^{\alpha } + \beta ^\alpha \varvec{v}^{\alpha } \; \mathrm{d}\varOmega (t), \end{aligned}$$

where $\varvec{\sigma }^{\alpha }$ is the Cauchy stress tensor for the $\alpha =s,f$, $\varvec{f}$ is a body force, $\hat{\varvec{p}}^{\alpha }$ are interaction forces representing frictional interactions between the solid and fluid (see 1) and $\beta ^\alpha$ is the constituent source term. Here $\beta ^s=0$ and $\beta ^f= \rho ^f g$. Applying Reynolds Transport Theorem, we rewrite the integral conservation law in differential form and obtain

38 $$\begin{aligned}&\frac{\partial (\hat{\rho }^\alpha \varvec{v}^{\alpha }) }{\partial t} + (\varvec{v}^\alpha \cdot \nabla ) (\hat{\rho }^\alpha \varvec{v}^{\alpha }) + \hat{\rho }^\alpha \varvec{v}^{\alpha } (\nabla \cdot \varvec{v}^\alpha ) \nonumber \\&\quad = \nabla \cdot \varvec{\sigma }^{\alpha } + \hat{\rho }^{\alpha }\varvec{f} + \hat{\varvec{p}}^{\alpha } + \beta ^\alpha \varvec{v}^{\alpha } \quad \text{ in }\;\varOmega (t). \end{aligned}$$

Expanding the LHS,

$$\begin{aligned} \begin{aligned}&\hat{\rho }^{\alpha } \left( \frac{\partial \varvec{v}^{\alpha } }{\partial t} + (\varvec{v}^\alpha \cdot \nabla ) \varvec{v}^{\alpha } \right) + \left( \frac{\partial \hat{\rho }^\alpha }{\partial t} + (\varvec{v}^\alpha \cdot \nabla ) \hat{\rho }^\alpha + \hat{\rho }^\alpha (\nabla \cdot \varvec{v}^\alpha ) \right) \varvec{v}^{\alpha } \\&\quad =\hat{\rho }^{\alpha } \frac{D\varvec{v}^{\alpha }}{Dt} + \left( \frac{\partial \hat{\rho }^\alpha }{\partial t} + \nabla \cdot (\hat{\rho }^\alpha \varvec{v}^\alpha ) \right) \varvec{v}^{\alpha }. \end{aligned} \end{aligned}$$

Using (34) and (35) to replace the second term above,

39 $$\begin{aligned} \hat{\rho }^\alpha \varvec{a}^\alpha = \nabla \cdot \varvec{\sigma }^\alpha + \hat{\rho }^\alpha \varvec{f} + \hat{\varvec{p}}^\alpha \quad \text{ in }\;\varOmega (t), \end{aligned}$$

where

40 $$\begin{aligned} \varvec{a}^s(\varvec{x},t)|_{\varvec{x}={\varvec{\chi }}(\varvec{X},t)}= & {} \frac{\partial ^{2}\varvec{\chi }(\varvec{X},t)}{\partial t^{2}} \,, \end{aligned}$$

41 $$\begin{aligned} \varvec{a}^f= & {} \frac{\partial \varvec{v}^f}{\partial t} + (\varvec{v}^f \cdot \nabla )\varvec{v}^f. \end{aligned}$$

### Constitutive relationships

Constitutive relationships for the interaction forces, permeability tensor and solid and fluid stress tensors are provided below. We choose expressions for the constitutive laws that appear frequently in the literature.

#### Interaction forces

The interaction force is given by

42 $$\begin{aligned} \hat{\varvec{p}}^{s}=-\hat{\varvec{p}}^{f}=-p\nabla \phi + \phi ^{2} \varvec{k}^{-1}\cdot (\varvec{v}^{f}-\varvec{v}^{s}), \end{aligned}$$

where $\varvec{k}$ is the (dynamic) permeability tensor and *p* is the fluid pressure [16]. The first term, $p \nabla \phi$, accounts for the pressure effect resulting from the variation of the section offered to the fluid flow, and the second term, $\phi ^{2} \varvec{k}\cdot (\varvec{v}^{f}-\varvec{v}^{s})$, describes the viscous resistance opposed by the shear stress to the fluid flow from the drag at the internal walls of the porous network. This particular choice for the interaction force means that the momentum balance for the fluid flow can later be reduced to the well known Darcy law.

#### Permeability tensor

The permeability tensor is given by

43 $$\begin{aligned} \varvec{k}=J^{-1} \varvec{F} \varvec{k}_{0}(\varvec{C}) \varvec{F}^{T} , \end{aligned}$$

where $\varvec{k}_{0}(\varvec{C})$ is the permeability in the reference configuration, which may be chosen to be some (nonlinear) function dependent on the deformation. Examples of deformation dependent permeability tensors for biological tissues can be found in [24, 34, 35]. A common isotropic assumption is

44 $$\begin{aligned} \varvec{k}=\kappa _{0}\varPi \left( J \right) \varvec{I}, \end{aligned}$$

where $\kappa _{0}$ is the permeability in the reference configuration and $\varPi \left( J \right)$ is some function dependent on the volume change. For example, in [34], the following isotropic constitutive law for the permeability of lung tissue is proposed

45 $$\begin{aligned} \varvec{k}=\kappa _{0} \left( J \frac{ \phi }{\phi _{0}} \right) ^{2/3}\varvec{I}, \end{aligned}$$

where $\kappa _{0}$ is the permeability in the reference configuration.

#### Solid stress tensor

The solid stress tensor is given by [8],

46 $$\begin{aligned} \varvec{\sigma }^{s} =\varvec{\sigma }^{s}_{e} - (1-\phi ) p \varvec{I}, \end{aligned}$$

where $\varvec{\sigma }^{s}_{e}$ is the effective stress tensor given by

47 $$\begin{aligned} \varvec{\sigma }^{s}_{e}=\frac{1}{J}\varvec{F}\cdot 2 \frac{\partial W(\varvec{C})}{\partial \varvec{C}} \cdot \varvec{F}^{T}. \end{aligned}$$

Here $W(\varvec{C})$ denotes a strain-energy law (hyperelastic Helmholtz energy functional) dependent on the deformation of the solid.

#### Fluid stress tensor

The fluid stress tensor can be written as [8],

48 $$\begin{aligned} \varvec{\sigma }^{f} =\varvec{\sigma }^{f}_{vis} - \phi p \varvec{I}, \end{aligned}$$

where $\varvec{\sigma }^{f}_{vis}$ denotes the viscous stress tensor of the fluid, given by

49 $$\begin{aligned} \varvec{\sigma }^{f}_{vis} = \mu _{f} \phi \left( (\nabla \varvec{v}_f) + (\nabla \varvec{v}_f)^{T} - \frac{2}{3}\nabla \cdot \varvec{v}_f \right) , \end{aligned}$$

where $\mu _{f}$ is the dynamic viscosity of the fluid.

### The general poroelasticity model

Summing the conservation laws for solid and fluid and applying the constitutive relations, the conservation of linear momentum for the mixture is

50 $$\begin{aligned} \hat{\rho }^{s}\varvec{a}^{s}+\hat{\rho }^{f}\varvec{a}^{f} =\nabla \cdot ( \varvec{\sigma }_{e}+\varvec{\sigma }_{vis}-p\varvec{I}) + \rho \varvec{f} \quad \text{ in } \; \varOmega (t). \end{aligned}$$

The momentum equation for the fluid flow alone is

51 $$\begin{aligned} \hat{\rho }^{f} \varvec{a}^f =\nabla \cdot ( \varvec{\sigma }_{vis}^f - \phi p \varvec{I}) + \hat{\rho }^{f}\varvec{f} + p \nabla \phi -\phi ^{2} \varvec{k}^{-1}(\varvec{v}^{f}-\varvec{v}^{s}) \quad \text{ in } \, \varOmega (t). \end{aligned}$$

We define the boundary $\partial \varOmega (t) = \varGamma _{d}(t) \cup \varGamma _{n}(t)$ for the mixture and $\partial \varOmega (t) = \varGamma _{p}(t) \cup \varGamma _{f}(t)$ for the fluid, with an outward pointing unit normal $\varvec{n}$. The problem for the mixture theory model is: Find $\varvec{\chi }(\varvec{X},t)$, $\varvec{v}^{f}(\varvec{x},t)$ and $p(\varvec{x},t)$ such that

52 $$\begin{aligned} \left. \begin{array}{cc} \begin{aligned} \hat{\rho }^{s} \varvec{a}^{s}+\hat{\rho }^{f} \varvec{a}^{f} &{}= \nabla \cdot ( \varvec{\sigma }_{e} + \varvec{\sigma }_{vis}-p\varvec{I}) + \rho \varvec{f} &{} \text{ in } \; \varOmega (t), \\ \hat{\rho }^{f}\varvec{a}^{f} &{}= \nabla \cdot ( \varvec{\sigma }_{vis}^{f}- \phi p \varvec{I}) + p \nabla \phi &{} \\ &{}\qquad - \phi ^2 \varvec{k}^{-1}(\varvec{v}^{f}-\varvec{v}^{s}) + \hat{\rho }^{f}\varvec{f} \quad &{}\text{ in } \; \varOmega (t), \\ \nabla \cdot ((1-\phi ) \varvec{v}^{s}) + \nabla \cdot (\phi \varvec{v}^f) &{}= g &{}\text{ in } \; \varOmega (t), \\ {\varvec{\chi }}(\varvec{X},t)|_{\varvec{X}={\varvec{\chi }}^{-1}(\varvec{x},t)} &{}= \varvec{X}+\varvec{u}_{D} &{}\; \text{ on } \; \varGamma _d(t), \\ (\varvec{\sigma }_{e}+\varvec{\sigma }_{vis}-p\varvec{I})\varvec{n} &{}= \varvec{t}_{N}, &{}\; \text{ on } \; \varGamma _n(t), \\ \varvec{v}^{f} &{}= \varvec{v}^{f}_{D} &{}\; \text{ on } \; \varGamma _f(t), \\ \varvec{n} \cdot \varvec{\sigma }_{vis}^{f} \cdot \varvec{n} - \phi p \varvec{I} \cdot \varvec{n} &{}= \varvec{s}_D &{}\; \text{ on } \; \varGamma _p(t), \\ \varvec{\chi }(\varvec{X},0) = \varvec{X} , \quad \varvec{v}^{s}(\varvec{X},0) &{}= {\varvec{v}^{s0}}, \quad \varvec{v}^{f}(\varvec{X},0) = {\varvec{v}^{f0}} &{}\text{ in } \; \varOmega (0). \end{aligned} \end{array} \right\} \end{aligned}$$

### Simplification of the model

We assume accelerations $\varvec{a}^\alpha$ and the viscous shear stress in the fluid $\varvec{\sigma }_{vis}^{f}$ are small, and define the fluid flux variable

53 $$\begin{aligned} \varvec{z}=\phi (\varvec{v}^{f}-\varvec{v}^{s}). \end{aligned}$$

The resulting problem is: Find $\varvec{\chi }(\varvec{X},t)$, $\varvec{z}(\varvec{x},t)$ and $p(\varvec{x},t)$ such that

54 $$\begin{aligned} \left. \begin{array}{cc} \begin{aligned} -\nabla \cdot ( \varvec{\sigma }_{e} -p\varvec{I}) &{}= \rho \varvec{f} \\ {\varvec{k}^{-1}\varvec{z}} + \nabla p &{}= \rho ^{f}\varvec{f} \\ \nabla \cdot (\varvec{v}^{s} + \varvec{z} ) &{}= g \\ {\varvec{\chi }}(\varvec{X},t)|_{\varvec{X}={\varvec{\chi }}^{-1}(\varvec{x},t)} &{}= \varvec{X}+\varvec{u}_{D} \\ (\varvec{\sigma }_{e}-p\varvec{I})\varvec{n} &{}= \varvec{t}_{N} \\ \varvec{z} \cdot \varvec{n} &{}= {q_{D}} \\ p &{}= p_{D} \\ \varvec{\chi }(\varvec{X},0) &{}= \varvec{X} \end{aligned}&{} \begin{aligned} &{}\text{ in } \; \varOmega (t), \\ &{}\text{ in } \; \varOmega (t), \\ &{}\text{ in } \; \varOmega (t), \\ &{}\text{ on } \; \varGamma _d(t), \\ &{}\text{ on } \; \varGamma _t(t), \\ &{}\text{ on } \; \varGamma _f(t), \\ &{}\text{ on } \; \varGamma _p(t), \\ &{}\text{ in } \; \varOmega (0). \end{aligned}\end{array} \right\} \end{aligned}$$

We note that, for example

55 $$\begin{aligned} \begin{aligned} \int _{\varOmega _{t}}\nabla _{\varvec{x}} \cdot \sigma (\varvec{x}) \; \mathrm{d}\varvec{x}&= \int _{\varOmega _{t}}\nabla _{\varvec{X}} \cdot \varvec{F}^{-1}\sigma (\varvec{\chi }(\varvec{X},t)) \; \mathrm{d}\varvec{x}\\&= \int _{\varOmega _{0}}\nabla _{\varvec{X}} \cdot \varvec{F}^{-1}J\sigma (\varvec{\chi }(\varvec{X},t)) \; \mathrm{d}\varvec{X} \\&= \int _{\varOmega _{0}}\nabla _{\varvec{X}} \cdot (\varvec{S}\varvec{F}^{T}) \; \mathrm{d}\varvec{X}, \end{aligned} \end{aligned}$$

where $\varvec{S}$ is the second Piola-Kirchoff stress tensor.

## Appendix 2: The fourth-order spatial tangent modulus tensor ${\varTheta }_{ijkl}$

The fourth-order spatial tangent modulus tensor ${\varTheta }_{ijkl}$ can be written as (in component form, see [9, section 5.3.2] and [25, section 6.6] )

56 $$\begin{aligned} {\varTheta }_{ijkl}= \frac{1}{J}F_{iI}F_{jJ}F_{kK}F_{lL}\mathbf {C}_{IJKL}, \end{aligned}$$

where ${\mathbf {C}}$ is the associated tangent modulus tensor in the reference configuration, given by

57 $$\begin{aligned} {\mathbf {C}}_{IJKL}= \frac{4 \partial ^{2} W}{\partial C_{IJ} \partial C_{KL} } + pJ \frac{\partial {C}^{-1}_{IJ}}{\partial {C}_{KL}}. \end{aligned}$$

For the numerical examples we have used the following Neo-Hookean strain-energy law

58 $$\begin{aligned} W(\varvec{C})=\frac{\mu }{2}(\text{ tr }(\varvec{C})-3)+\frac{\varLambda }{4}(J^{2}-1)-(\mu +\frac{\varLambda }{2})\text{ ln }(J-1+\phi _{0}). \end{aligned}$$

Thus, the resulting effective stress tensor is given by

59 $$\begin{aligned} \varvec{\sigma }_{e}=\frac{\varLambda }{2}\left( J-\frac{1}{J-1+\phi _{0}}\right) \varvec{I} + {\mu }\left( \frac{\varvec{C}^{T}}{J} - \frac{\varvec{I}}{J-1+\phi _{0}}\right) , \end{aligned}$$

and the spatial tangent modulus tensor is given as

60 $$\begin{aligned} \varvec{\Theta }=\varvec{\Theta }_{e} + p ( \varvec{I} \otimes \varvec{I} - 2 \mathcal {Z}), \end{aligned}$$

where

61 $$\begin{aligned} \varvec{\Theta }_{e}= & {} \left[ \varLambda J-2\mu \left( \frac{1}{2(J-1+\phi _{0})} -\frac{J}{2(J-1+\phi _{0})^{2}} \right) \right] \varvec{I}\otimes \varvec{I}\nonumber \\&+ \left[ \frac{2 \mu }{J-1+\phi _{0}} - \varLambda (J-\frac{1}{J-1+\phi _{0}} ) \right] \mathcal {B}, \end{aligned}$$

and

62 $$\begin{aligned} \mathcal {B}_{ijkl}=\frac{1}{2}(\delta _{ik}\delta _{jl} + \delta _{il}\delta _{jk}), \quad \mathcal {Z}_{ijkl}= \delta _{ik}\delta _{jl}, \quad \varvec{I} \otimes \varvec{I}= \delta _{ij}\delta _{kl}. \end{aligned}$$

See [9, chapter 5] and [57, chapter 3] for further details.

To simplify the implementation of the spatial tangent modulus we make use of matrix Voigt notation. The matrix form of $\mathbf {\beta }$ is given by $\varvec{D}$, which can be written as (see [9, section 7.4.2])

63 $$\begin{aligned} \varvec{D}=\frac{1}{2} \begin{pmatrix} 2\varvec{\Theta }_{1111}&{}2\varvec{\Theta }_{1122}&{}2\varvec{\Theta }_{1133}&{} \varvec{\Theta }_{1112}+\varvec{\Theta }_{1121}&{}\varvec{\Theta }_{1113}+\varvec{\Theta }_{1131}&{}\varvec{\Theta }_{1123}+\varvec{\Theta }_{1132}\\ &{} 2\varvec{\Theta }_{2222} &{} 2\varvec{\Theta }_{2233} &{}\varvec{\Theta }_{2212}+\varvec{\Theta }_{2221} &{}\varvec{\Theta }_{2213}+\varvec{\Theta }_{2231}&{} \varvec{\Theta }_{2223}+\varvec{\Theta }_{2232} \\ &{} &{} 2 \varvec{\Theta }_{3333} &{} \varvec{\Theta }_{3312}+\varvec{\Theta }_{3321} &{} \varvec{\Theta }_{3313}+\varvec{\Theta }_{3331} &{} \varvec{\Theta }_{3323}+\varvec{\Theta }_{3332} \\ &{} &{} &{} \varvec{\Theta }_{1212}+\varvec{\Theta }_{1221} &{} \varvec{\Theta }_{1213}+\varvec{\Theta }_{1231} &{} \varvec{\Theta }_{1223} + \varvec{\Theta }_{1232} \\ &{} \text{ sym. } &{} &{} &{} \varvec{\Theta }_{1313}+\varvec{\Theta }_{1331} &{} \varvec{\Theta }_{1323}+\varvec{\Theta }_{1332} \\ &{} &{} &{} &{} &{} \varvec{\Theta }_{2323} + \varvec{\Theta }_{2332} \\ \end{pmatrix}. \end{aligned}$$

We also make use of the following implementation friendly matrix notation for $\nabla ^{S}\varvec{\phi }_{k}$

64 $$\begin{aligned} \varvec{E}_{k}= \begin{bmatrix} {\phi }_{k,1}&\quad 0&\quad 0\\ 0&\quad {\phi }_{k,2}&\quad 0\\ 0&\quad 0&\quad {\phi }_{k,3}\\ \phi _{k,2}&\quad \phi _{k,1}&\quad 0\\ 0&\quad {\phi }_{k,3}&\quad {\phi }_{k,2}\\ {\phi }_{k,3}&\quad 0&\quad {\phi }_{k,1}\\ \end{bmatrix}. \end{aligned}$$
